# Enhanced machine learning predictive modeling for delirium in elderly ICU patients with COPD and respiratory failure: A retrospective study based on MIMIC-IV

**DOI:** 10.1371/journal.pone.0319297

**Published:** 2025-03-20

**Authors:** Zong-bi Wu, You-li Jiang, Shuai-shuai Li, Ao Li

**Affiliations:** 1 Nursing Department, Shenzhen Traditional Chinese Medicine Hospital (The Fourth Clinical Medical School of Guangzhou University of Chinese Medicine), Shenzhen, China; 2 Department of Neurology, People’s Hospital of Longhua, Shenzhen, China; 3 Clinical Nursing Teaching and Research Section, The Second Xiangya Hospital of Central South University, Changsha, China; Azienda Ospedaliero Universitaria Careggi, ITALY

## Abstract

**Background and objective:**

Elderly patients with Chronic obstructive pulmonary disease (COPD) and respiratory failure admitted to the intensive care unit (ICU) have a poor prognosis, and the occurrence of delirium further worsens outcomes and increases hospitalization costs. This study aimed to develop a predictive model for delirium in this patient population and identify associated risk factors

**Methods:**

Data for the machine learning model were obtained from the MIMIC-IV database. Feature variable screening was conducted using Lasso regression and the best subset method. Four models—K-nearest neighbor, random forest, logistic regression, and extreme gradient boosting (XGBoost)—were trained and optimized to predict delirium risk. The stability of the model is evaluated using ten-fold cross validation and the effectiveness of the model on the validation set is evaluated using accuracy, F1 score, precision and recall. The SHapley Additive exPlanations (SHAP) method was used to explain the importance of each variable in the model.

**Results:**

A total of 1,155 patients admitted to the intensive care unit between 2008 and 2019 were included in the study, with a delirium incidence of 12.9% (149/1,155). Among the four ML models evaluated, the XGBoost model demonstrated the best discriminative ability. In the validation set, it achieved an AUC of 0.932, indicating superior performance with high accuracy, precision, recall, and F1 scores of 0.891, 0.839, 0.795, and 0.810, respectively. Key features identified through SHAP analysis included the Glasgow Coma Scale (GCS) verbal score, length of hospital stay, mean SpO₂ on the first day of ICU admission, Modification of Diet in Renal Disease (MDRD) equation score, mean diastolic blood pressure, GCS motor score, gender, and duration of noninvasive ventilation. These findings provide valuable insights for individualized risk management.

**Conclusions:**

The developed prediction model effectively predicts the occurrence of delirium in elderly COPD patients with respiratory failure in the ICU. This model can assist clinical decision-making, potentially improving patient outcomes and reducing healthcare costs.

## 1. Introduction

Chronic obstructive pulmonary disease (COPD) represents a significant public health challenge in China, characterized by persistent respiratory symptoms and airflow obstruction. The prevalence of COPD is approximately 11.6% among the Chinese population aged 40 and above [1]. The disease follows a chronic and progressive course, often complicated by comorbidities [2]. Respiratory failure is a common and severe complication of COPD, substantially impacting patient prognosis and associated with a mortality rate of 62% in those affected [3]. These critically ill patients require intensive monitoring and prompt intervention, requiring management in the intensive care unit (ICU) instead of general wards [4]. Delirium, characterized by fluctuating impairments in attention, consciousness, and cognition, was found to have an incidence rate of 21.94% in patients with COPD complicated by respiratory failure[5]. The condition is associated with severe consequences, including postoperative falls, prolonged hospital stays, poor functional recovery, increased readmissions, non-family discharges, long-term cognitive decline, and higher mortality rates [6–8]. Xu et al. demonstrated that delirium serves as an independent risk factor for both long-term and short-term mortality in COPD patients following ICU admission [9]. Furthermore, Kerber et al. identified an association between COPD and delirium, highlighting it as an independent predictor of unplanned extubation in the ICU. Therefore, special attention should be paid to delirium in COPD patients in the ICU [10]. Despite its significant impact, pharmacologic treatment options for delirium remain limited [11]. However, early identification of at-risk patients and timely intervention can improve outcomes for those with respiratory failure who develop delirium. Previous studies have identified invasive mechanical ventilation, sedative medications, and protective restraints as significant risk factors for delirium [12], yet these interventions are essential in the ICU setting. Early recognition and intervention of delirium remain challenging for healthcare providers [13]. Machine learning, a critical component of artificial intelligence, has shown promise in predicting clinical outcomes by utilizing large clinical datasets for predictive tasks such as regression and classification. Its applications in medicine have expanded rapidly, demonstrating good predictive accuracy in areas such as blood-borne infections in ICUs, stress injuries, and sepsis prognosis [14–17]. However, research on early warning models for ICU delirium in COPD patients with respiratory failure remains limited. In an earlier study, Wassenaar et al. employed multiple logistic regression to develop the E-PRE-DELIRIC predictive model for delirium in ICU patients, achieving an AUC of 0.75 (95% CI 0.71-0.79) in the validation dataset [18]. With the increasing application of machine learning techniques in the medical field, more delirium prediction models are being developed for specific target populations. Ren et al. utilized a random forest algorithm to develop a delirium prediction model for patients with extensive burns post-surgery, achieving robust performance in external validation, with an accuracy of 77.12%, sensitivity of 67.74%, and specificity of 80.46% [19]. Lee et al. developed a prediction model for delirium following non-cardiac surgery using XGBoost, incorporating 5 variables, and achieved an AUROC of 0.867 (0.845–0.877) in external validation [20]. These studies underscore the significant potential of machine learning in facilitating early diagnosis and prevention of clinical diseases. Studies have shown that chronic noninvasive mechanical ventilation (NIV) has become one of the mainstay treatments for patients with COPD with respiratory failure [21]. However, Zhang et al. showed that delirium is associated with an increase in numerous adverse outcomes (NIV failure, ICU mortality, and in-hospital mortality), and therefore, early prevention and treatment of delirium in this population is necessary to improve the long-term prognosis of patients [22].

Considering the high prevalence of delirium among COPD patients with respiratory failure in the ICU and its associated poor prognosis, there is a critical need for a reliable early warning model. This study aims to develop a machine learning-based risk prediction model for delirium in elderly COPD patients with respiratory failure in the ICU and to identify the key risk factors for delirium in this population. Utilizing extensive and reliable clinical data from public databases, our results are intended to serve as a valuable tool for clinicians to aid in decision-making.

## 2. Materials and methods

### 2.1. Data source

This study utilized data from the publicly available MIMIC-IV v2.0 database, which contains clinical records from over 190,000 patients admitted to Beth Israel Deaconess Medical Center (BIDMC) at Harvard Medical School, covering 450,000 hospitalizations from 2008 to 2019. The database includes comprehensive information on patient demographics, laboratory tests, medications, vital signs, surgical procedures, disease diagnoses, medication management, and follow-up survival status. In order to protect patient privacy and comply with laws and regulations, the data in the MIMIC database have been anonymized, including the removal of any information that could identify the patient.

### 2.2. Participants

**Fig 1:**
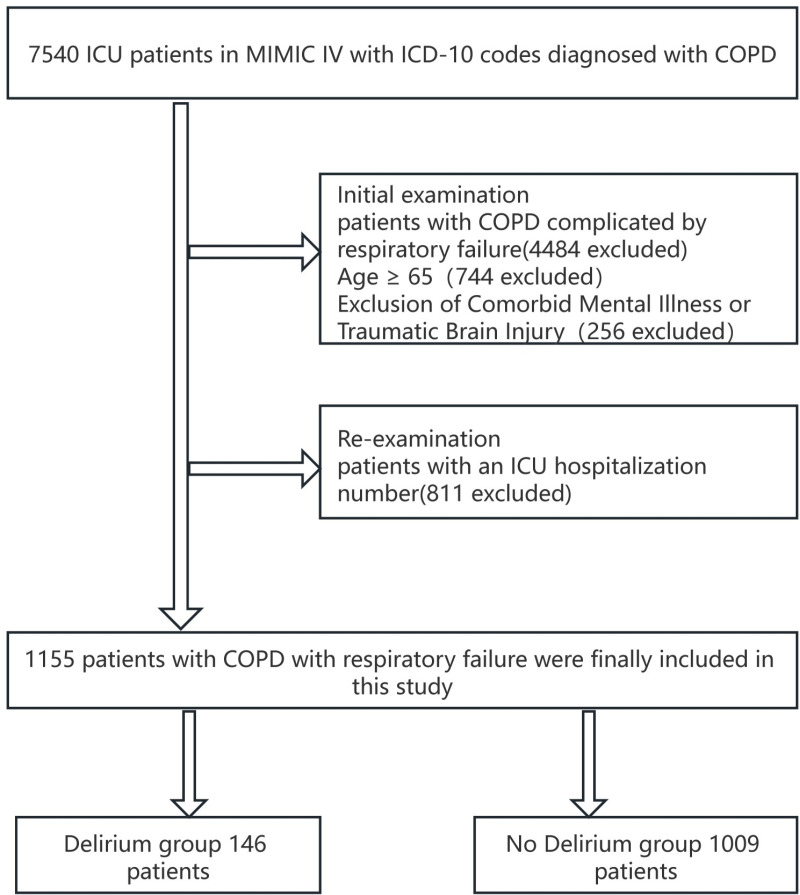
Flow chart.

This study included patients aged ≥ 65 years admitted to the ICU with COPD complicated by respiratory failure. All included patients had available data on vital signs, laboratory tests, and the type and duration of respiratory support. As depicted in Fig 1, patients under 65 years and those with psychiatric illness or traumatic brain injury diagnoses were excluded. We extracted records from the first ICU stay and categorized patients into delirium and non-delirium groups based on ICD-9 or ICD-10 diagnostic codes for delirium during their stay.

### 2.3. Variable selection

We installed the MIMIC database using SQL Server 2012 and utilized SQL queries to extract the demographics, clinical characteristics, and laboratory findings of elderly patients with COPD complicated by delirium within 24 hours of ICU admission. Variables were selected during data extraction based on prior studies and clinical expertise, and predictors included (a) demographic information: age, gender, marital status, insurance, race, and weight. (b) Clinical scores on the first day in the ICU: SOFA, APS III, SIRS, and GCS scores for each dimension. (c) Vital signs on the first day in the ICU: heart rate, systolic blood pressure (SBP), diastolic blood pressure (DBP), mean arterial pressure (MAP), respiratory rate, SpO₂, and urine output. (d) Maximum and minimum values of laboratory findings on the first day of ICU admission: PH, PO^2^, PCO^2^, BE, Total CO^2^, glomerular filtration rate (GFR, calculated using the MDRD equation), Platelets Wbc (White Blood Cells), anion gap (AG), HCO^3^, blood urea nitrogen (Bun), Calcium (blood urea nitrogen), and Bun (blood urea nitrogen). Urea Nitrogen), Calcium, International Normalized Ratio (INR), Prothrombin Time (PT), Partial Thromboplastin Time (PTT), Hematocrit (Hematocrit), Hemoglobin (Hemoglobin), and Glucose averages.(e) Maximum and minimum serum creatinine (Scr) values during hospitalization. Almost all patients undergo a series of laboratory tests, recording of vital signs, and monitoring within 24 hours of admission to the ICU, and are also evaluated by professionals using scales. We selected variables that were generalized, as well as variables with fewer missing values in MIMIC IV, in order to seek as comprehensive a variable inclusion and screening as possible.

### 2.4. Statistical analysis

The normality of continuous variables was assessed using the Shapiro-Wilk test. Normally distributed variables were expressed as mean and standard deviation, while non-normally distributed variables were expressed as median (interquartile range, IQR). Comparisons between two groups were performed using Student’s t-test or the Wilcoxon rank-sum test as appropriate. Categorical variables were presented as counts and percentages, and comparisons between groups were made using the chi-square test. Variables with more than 10% missing values were excluded, and remaining missing values were imputed using random forest-based multiple imputation. To address the class imbalance between negative and positive groups, the Synthetic Minority Over-sampling Technique (SMOTE) was employed to balance the dataset. The dataset was split into training and validation sets in an 80/20 ratio. Feature selection was performed using Lasso regression and the best subset method, with the selected variables detailed in the S1 File. Two feature combinations were used to train the models. Four machine learning algorithms were employed: K Nearest Neighbors (KNN), Random Forest (RF), Extreme Gradient Boosting (XGBoost), and traditional Logistic Regression. Model performance was evaluated using ten-fold cross-validation, with comparisons based on the area under the receiver operating characteristic curve (AUC-ROC). Model performance on the validation set was evaluated using four metrics: accuracy, F1 score, precision, and recall. The SHAP technique [23] was utilized to interpret the models’ variables. All analyses were conducted using R version 4.3.2.

## 3. Results

### 3.1. Patients characteristics

A total of 1,155 patients were identified and included from the publicly available MIMIC-IV 2.0 database. The screening process is detailed in Fig 1. Variables with more than 10% missing data, including myocardial markers, were excluded. Sixty-five predictor variables were selected for model construction, including age, weight, length of hospitalization, clinical scores, vital signs, ventilation mode, and laboratory tests. Patients were divided into two groups: a delirium group (n = 146) and a non-delirium group (n = 1009). Baseline characteristics are summarized in S1 Table. Patients in the delirium group were older on average (78.5 ±  7.6 vs. 76.9 ±  7.8 years) and a higher proportion of males (59.6% vs. 40.4%). Significant differences were observed between the groups in terms of length of hospitalization and ICU stay (P < 0.001), as well as SOFA and APS III scores (P < 0.05). No significant differences were found in comorbidities, marital status, weight, and insurance (P > 0.05).

### 3.2. Feature screening and model training

**Fig 2:**
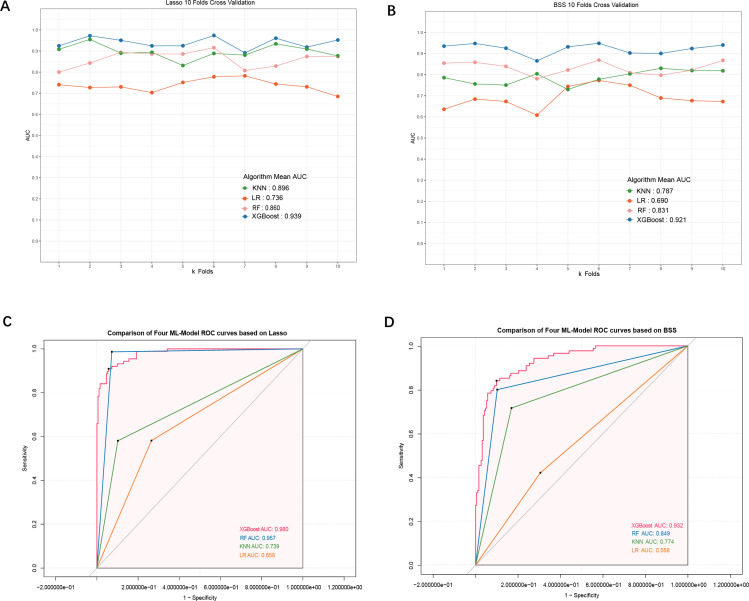
Comparison of Machine Learning Models. (A) Ten-fold cross-validation mean AUC for KNN, RF, LR, XGBoost (Lasso regression); (B) Ten-fold cross-validation mean AUC for KNN, RF, LR, XGBoost (Best Subset Selection); (C) AUC-ROC curves for KNN, RF, LR, XGBoost (Lasso regression); (D) AUC-ROC curves for KNN, RF, LR, XGBoost (Best Subset Selection).

**Fig 3:**
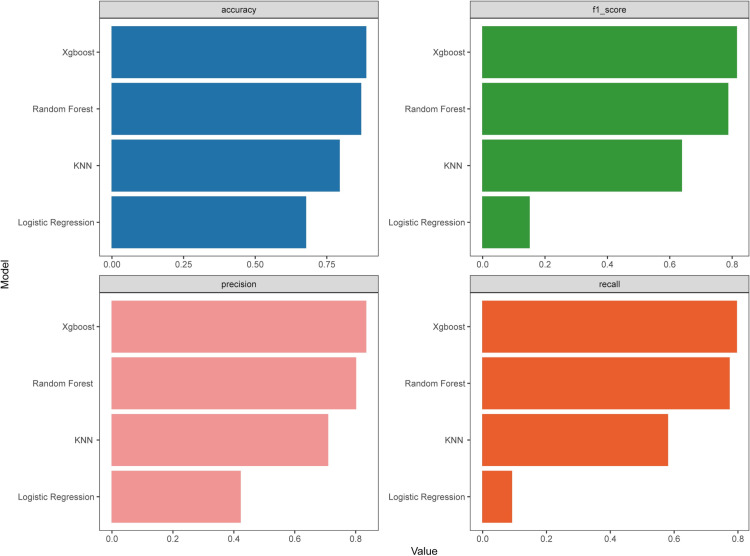
Comparison of Accuracy, F1 Score, Precision, and Recall of Four Machine Learning Models on the Test Dataset.

The feature screening process, detailed in the Supplementary Material, employed two methods: LASSO and the optimal subset method. These methods identified 34 variables and 9 combinations of variables. During model training, the GCS variable, identified as the least important, was excluded. The 8-variable XGBoost model demonstrated improved performance and reduced complexity compared to the 9-variable model. Therefore, we optimized the subset regression by incorporating 8 variables. Based on these results, we constructed the dataset and partitioned the data. Four algorithms—KNN, RF, XGBoost, and LR—were applied for model training and parameter optimization on the training set. Ten-fold cross-validation was used to evaluate the models (Fig 2A, B). The average AUCs of the four models on the test set were 0.787, 0.690, 0.831, and 0.921, respectively, with the XGBoost model achieving the highest AUC and demonstrating superior stability. Additionally, on the validation set, the XGBoost model showed the highest AUC (Fig 2C, D), along with superior performance in terms of accuracy (0.891), F1 score (0.810), precision (0.839), and recall (0.795) (Fig 3).

### 3.3. Model selection and feature interpretation

**Fig 4:**
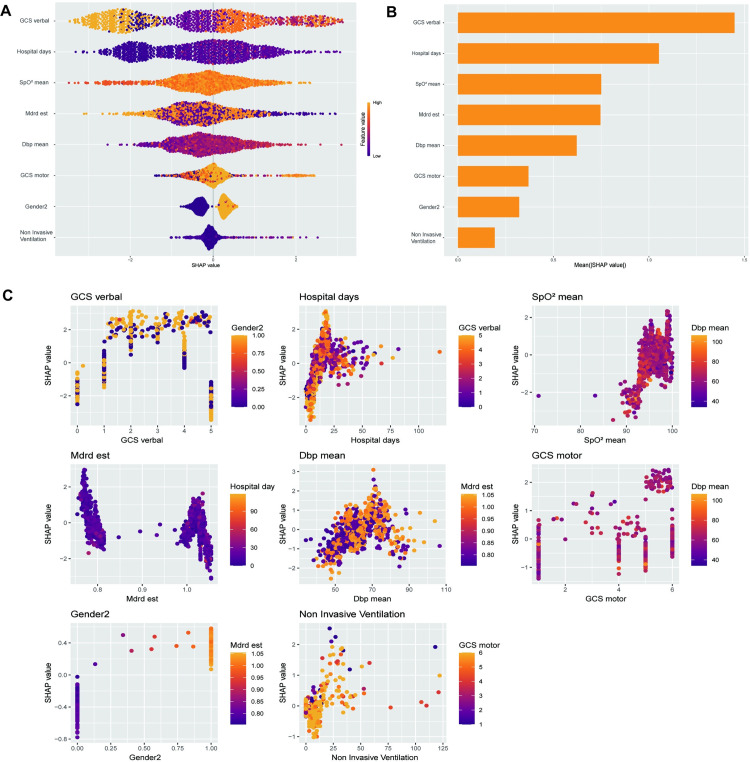
SHAP-based Model Interpretation. (A) SHAP summary dot plot. Higher SHAP values indicate increased likelihood of delirium. Dots represent patients’ SHAP values, colored by feature value (yellow for higher, purple for lower); (B) Importance ranking of variables in the XGBoost model. Key features include mean diastolic blood pressure (Dbp min) and glomerular filtration rate (MDRD est). (C) SHAP value plots for various features in a model. SHAP values elucidate the impact of each feature on the model’s output. Each subplot illustrates the relationship between a specific feature and its SHAP values, with additional feature values represented by color gradients.

**Fig 5:**
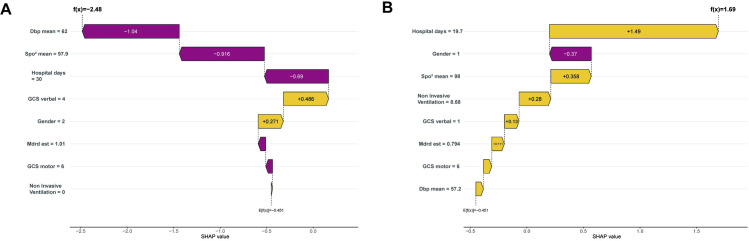
Interpretation of the local model using the SHAP method, including waterfall plots and the evolution of each characteristic’s contribution to individual risk Older adults categorized by low (A) or high (B) risk of developing delirium: ‘A’ indicates that the individual is not classified under the ‘delirium’ category, whereas ‘B’ indicates that the individual is classified under the ‘delirium’ category.

**Fig 6:**
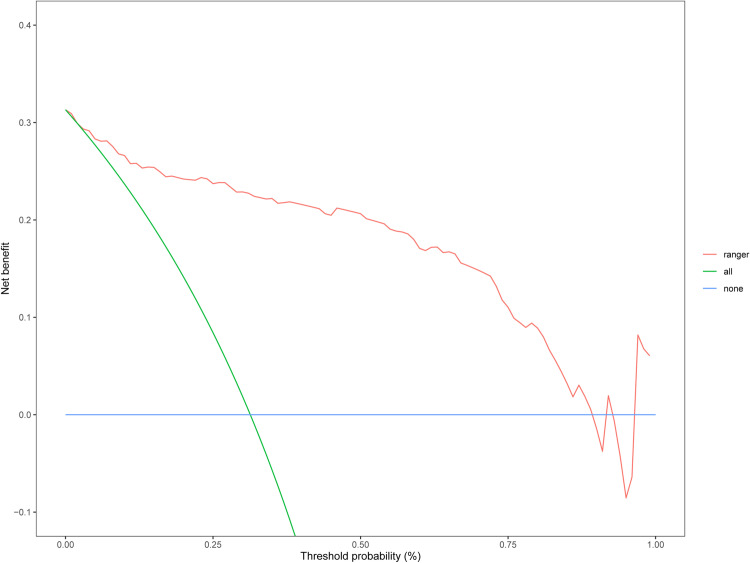
Decision Curve Analysis (DCA) of the Predictive Model.

Following model comparisons, the XGBoost model, developed using the optimal subset method, was selected for feature optimization. SHAP analysis was employed for global model interpretation (Fig 4A), providing a ranking of feature importance (Fig 4B). The results indicated that GCS Verbal, length of hospital stay, mean SpO2, mean DBP, and the MDRD equation were the top five contributors to the model. Dependency plots for individual model features illustrate their interactions. The SHAP values for the GCS verbal score (Glasgow Coma Scale verbal response) were distributed across its range. The color gradient represents gender, with darker colors indicating females and lighter colors indicating males, suggesting a potential gender-related effect on GCS verbal SHAP values. SHAP values for hospital days display a positive trend, indicating that longer hospital stays generally elevate the model’s prediction. The color gradient denotes GCS verbal, implying that the Glasgow Coma Scale verbal score also affects the SHAP values for hospital days. Furthermore, SHAP was employed for individual-level local interpretation, as depicted in Fig 5A and B. This individualized risk assessment elucidates the model’s capacity to predict delirium risk. A low-risk patient (predicted risk score: -2.48) was primarily influenced by factors such as mean diastolic blood pressure and mean finger pulse oximetry. Conversely, a high-risk patient (predicted risk score: 1.69) was primarily influenced by hospital length of stay and gender. This interpretability offers valuable insights for targeted risk management. Decision curve analysis (DCA) of the test dataset demonstrated that interventions based on the predictive modeling yielded favorable results (Fig 6).

## 4. Discussion

This study developed a predictive model for delirium in elderly COPD patients with respiratory failure in ICUs using MIMIC-IV data. Our model demonstrated high accuracy and reliability on the validation set. While the reduced model showed a slight decline in performance compared to the 34-variable model, its lower complexity significantly reduced implementation costs and improved generalizability, making the model more practical for clinical use. We used SHAP visualization techniques to interpret and rank the importance of each variable in the model. This predictive model can be seamlessly integrated into existing ICU workflows, allowing healthcare providers to quickly identify high-risk patients and implement preventive measures, ultimately improving patient outcomes and reducing healthcare costs.

We employed two feature selection techniques: Lasso regression and optimal subset regression. Lasso regression included more variables and yielded excellent predictive accuracy, while optimal subset regression achieved similar accuracy with fewer variables. Among the tested models (KNN, RF, and LR), XGBoost achieved the highest performance across all evaluation metrics. This highlights XGBoost’s suitability for this task, particularly in handling complex, non-linear relationships typical in ICU settings. The effectiveness of both Lasso and optimal subset regression validates our approach, ensuring high predictive power and computational efficiency.

Despite the significant advantages of machine learning, most models function as black boxes, making them difficult to interpret using traditional linear methods and raising concerns among clinicians. SHAP offers a means for model interpretation. The SHAP values quantify the contribution of each feature, which helps the clinical staff to better understand the predictive model and intervene accordingly [24]. We mitigated this challenge by employing SHAP for both global and local interpretation of the XGBoost model. Furthermore, we identified GCS verbal score, length of hospitalization, mean SpO2 on the first day, MDRD equation, mean diastolic blood pressure on the first day, GCS motor score, gender, and duration of noninvasive ventilation as key predictors of delirium in COPD patients with respiratory failure. Our findings are consistent with previous studies that identified hospital length of stay, GCS score, and noninvasive ventilation as key risk factors for delirium [25,26]. Notably, the selected variables—excluding laboratory tests—are readily available in routine clinical practice, lowering implementation barriers and improving generalizability. Focusing on accessible clinical data allows our model to be seamlessly integrated into ICU workflows, facilitating timely and cost-effective interventions. This approach is consistent with existing research, which shows that practical models based on routine clinical variables can achieve high predictive accuracy and improve patient outcomes. Future research should explore the model’s applicability across different patient subgroups, including various age ranges, genders, and those with different comorbidities, to further validate its generalizability.

Studies specifically addressing delirium in elderly COPD patients with respiratory failure are limited. Li and colleagues identified age, BMI, hypertension, APACHE II score, CPOT, sedation, and PaO^2^ as key factors for delirium in this population [5]. Although valuable, their study’s traditional analytical approach limits clinical applicability due to lower predictive precision. Our study builds on this groundwork by incorporating advanced machine learning techniques, enhancing the accuracy of delirium predictions. Leveraging these risk factors within an advanced machine learning framework, our model enhances predictive performance and clinical utility. Despite numerous studies developing prediction models for the risk of delirium in ICU patients using machine learning, most models have performed inadequately. Kim et al. developed a basic prediction model for ICU patients, achieving an AUROC of 0.779 (CI 0.748–0.811) in the test set [27], whereas our prediction model achieved an AUROC of 0.932 in the validation set. This discrepancy may be due to individual variability among ICU patients, who often present with complex conditions and receive diverse treatments. Focusing on specific disease subgroups may improve model predictive performance. Zhang et al. developed a prediction model for the risk of postoperative delirium in patients with degenerative spine disease using nine algorithms. The results indicated that the XGBoost algorithm exhibited the best performance, with an AUC of 0.879 (CI: 0.819-0.940) [28], similar to our findings. This underscores the superiority of the XGBoost algorithm for constructing predictive models. XGBoost is widely used in real-world applications due to its outstanding performance, robustness, regularization, and interpretability [29]. This methodological advancement overcomes previous study limitations and enhances the practical applicability of delirium risk assessment in ICUs, potentially enabling earlier and more effective interventions. Huang A. A [30] performed modeling on data from the England National Health Services Heart Disease Prediction Cohort using four algorithms, namely XGBoost, Random Forest, Artificial Neural Network, and Adaptive Boosting, while the XGBoost algorithm stood out and the AUROC of the constructed model ranged from 0.771 to 0.947, showing its strong potential, similar to our study.

The PIPRA tool developed by Dodsworth et al. achieved an AUC of 0.74 (0.68–0.80) in external validation for predicting postoperative delirium (POD) in elderly patients [31]. Similarly, Song et al.‘s model achieved an AUC of 0.71 for POD [32]. Our model, with an AUC of 0.921, surpasses these benchmarks, particularly for ICU patients with COPD and respiratory failure. This demonstrates our model’s effectiveness and reliability, And the previously unidentified predictive model for the risk of delirium in patients with COPD combined with respiratory failure provides a valuable tool for ICU providers in this field, to improve the prognosis of patients through timely intervention.

Notably, our final model included the patient’s first-day GCS verbal and motor scores. The GCS score, widely used to assess patient consciousness, is relevant as delirium affects consciousness. Similarly, Wang et al. developed a predictive model for delirium occurrence in ICU patients within 48 hours, the final incorporated features included the GCS score on the first day, which demonstrated the importance of assessing the GCS score within 24h of ICU admission in the early identification of delirium [33,34]. The study by Tang et al [35] also provided early prediction of delirium in elderly ICU patients in MIMIC IV and showed that GCS score, mechanical ventilation, and sedation were the top three most important influencing features, again illustrating the importance of GCS in the early recognition of delirium. Bodien et al. noted that the total GCS score might not accurately reflect consciousness levels and recommended considering individual subscale behaviors [36]. Our study reinforces this perspective by highlighting the critical role of GCS subscales, particularly the GCS verbal score, in delirium prediction, thereby simplifying the assessment process and enhancing its practicality.

In this study, XGBoost demonstrated superior predictive ability and accuracy compared to other algorithms. Classical logistic regression performed relatively poorly. Conversely, a study by Song et al. found that logistic regression outperformed complex machine learning algorithms in predicting postoperative delirium [37], differing from our findings. These differences may be due to variations in variable characteristics and data distribution. Feng et al. observed similar findings, noting that machine learning algorithms excel in predicting outcomes from large, complex datasets [38]. However, the black-box nature of the XGBoost model may make it less intuitive compared to traditional predictive models, and although the SHAP methodology has significantly reduced this disadvantage, it may still not be recognized by traditional healthcare practitioners. Thus, it’s crucial to apply various training strategies and select appropriate algorithms based on evaluation metrics when constructing risk prediction models.

This study has several limitations. First, this is a retrospective study. Although our model performed well on an internal validation set using the MIMIC dataset, the data originate from the United States and lack external validation across diverse populations. Therefore, validation of its actual predictive effect in a prospective cohort is needed. Second, our inclusion of vital signs and laboratory results was limited to within 24 hours of the patient’s admission to the intensive care unit. There may be some bias between the data due to differences in techniques and methods used by different personnel to collect these data, affecting the effectiveness of application in the clinic. Furthermore, patient indicators change over the course of hospitalization, which was not accounted for in our study. Future advancements in hospital technology may enable dynamic assessment of delirium risk based on hospitalization duration, allowing for tailored intervention strategies at different stages.

## 5. Conclusion

Our optimized XGBoost model demonstrated high accuracy and reliability, utilizing key clinical variables readily available in routine care. This practical and generalizable model can be effectively integrated into ICU workflows, enabling timely and cost-effective interventions. Leveraging advanced machine learning techniques, our study addresses the limitations of previous research and enhances clinical decision-making and patient outcomes. Future research should prioritize external validation across diverse settings and investigate additional predictive factors to enhance the model’s applicability. Overall, our study provides a valuable tool for the early identification and intervention of delirium, with the goal of improving patient outcomes and reducing healthcare costs.

## Supporting information

S1 Table Characteristics of delirium and non-delirium patients in the dataset of the MIMIC-IV database. (PDF)

S1 File Variable screening process (Lasso with best subset regression) (PDF)
