# Supplementary material for: Enhanced machine learning predictive modeling for delirium in elderly ICU patients with COPD and respiratory failure: A retrospective study based on MIMIC-IV
Source: PLoS One. 2025 Mar 20;20(3):e0319297. doi: 10.1371/journal.pone.0319297 (PMC11925466; doi:10.1371/journal.pone.0319297)
Supplement: S1 Table — (PDF) [file pone.0319297.s001.pdf]

Table S1 Characteristics of delirium and non-delirium patients in the dataset of the MIMIC-IV database

Variables	Levels	No delirium, n=1009 (%)	Delirium, n=146 (%)	*P-value*
Age (mean ± SE)		76.9 ± 7.8	78.5 ± 7.6	0.017
Gender	Female	511 (50.6)	59 (40.4)	0.026
	Male	498 (49.4)	87 (59.6)	
Marital Status	Married	556 (55.1)	71 (48.6)	0.391
	Divorced	87 (8.6)	14 (9.6)	
	Single	176 (17.4)	33 (22.6)	
	Widowed	190 (18.8)	28 (19.2)	
Insurance	Medicare	689 (68.3)	109 (74.7)	0.291
	Medicaid	11 (1.1)	1 (0.7)	
	Others	309 (30.6)	36 (24.7)	
Race	White	679 (67.3)	102 (69.9)	0.772
	Black	94 (9.3)	10 (6.8)	
	Hispanic	23 (2.3)	4 (2.7)	
	Others	213 (21.1)	30 (20.5)	
Weight (kg)	Median (IQR)	76.80 (62.70, 93.00)	75.45 (62.40, 96.50)	0.993
Hospital Days	Median (IQR)	9.00 (5.00, 16.00)	15.00 (10.00, 21.00)	<0.001
ICU Days	Median (IQR)	3.00 (1.00 to 6.00)	4.00 (2.00, 9.00)	<0.001
SOFA	Median (IQR)	6.00 (3.00 to 9.00)	7.00 (4.00 to 10.00)	0.031
APS III	Median (IQR)	50.00 (38.00, 66.00)	55.00 (43.00, 72.00)	0.031
SIRS	Median (IQR)	3.00 (2.00, 3.00)	3.00 (2.00 to 3.00)	0.653
**GCS Score**				
Verbal	Median (IQR)	4.00 (1.00, 5.00)	4.00 (1.00, 4.00)	0.002
Eyes	Median (IQR)	3.00 (3.00, 4.00)	3.00 (2.00, 4.00)	0.141
Motor	Median (IQR)	6.00 (5.00, 6.00)	6.00 (5.00, 6.00)	0.873
Renal Disease	No	639 (63.3)	81 (55.5)	0.082

	Yes	370 (36.7)	65 (44.5)	
Malignant Cancer	No	835 (82.8)	121 (82.9)	1.000
	Yes	174 (17.2)	25 (17.1)	
Peripheral Vascular Disease	No	833 (82.6)	117 (80.1)	0.549
	Yes	176 (17.4)	29 (19.9)	
Peptic Ulcer Disease	No	983 (97.4)	140 (95.9)	0.432
	Yes	26 (2.6)	6 (4.1)	
Diabetes	No	795 (78.8)	111 (76)	0.515
	Yes	214 (21.2)	35 (24)	
Severe Liver Disease	No	980 (97.1)	144 (98.6)	0.437
	Yes	29 (2.9)	2 (1.4)	
CKD	No	639 (63.3)	81 (55.5)	0.082
	Yes	370 (36.7)	65 (44.5)	
**Ventilation**				
HFNC, hours	Median (IQR)	0.00 (0.00, 0.00)	0.00 (0.00, 0.00)	0.383
Non-Invasive Vent, hours	Median (IQR)	0.00 (0.00, 0.00)	0.00 (0.00, 0.00)	0.157
Supplemental Oxygen, hours	Median (IQR)	22.00 (8.00, 45.00)	32.50 (13.00, 62.00)	0.002
Invasive Vent, hours	Median (IQR)	0.00 (0.00, 30.00)	10.50 (0.00, 46.00)	0.017
Tracheostomy, hours	Median (IQR)	0.00 (0.00, 0.00)	0.00 (0.00, 0.00)	0.022
**Vital Signs**				
Heart rates mean	Median (IQR)	86.40 (75.32, 99.26)	85.79 (75.35, 98.85)	0.811
Sbp mean	Median (IQR)	114.50 (105.68,126.74)	115.85 (105.80, 127.04)	0.460
Dbp mean	Median (IQR)	61.47 (55.17, 68.44)	62.16 (56.49, 69.50)	0.267
Mbp mean	Median (IQR)	75.86 (70.53, 83.24)	77.51 (71.15, 84.40)	0.155
Resp rate mean	Median (IQR)	20.52 (18.36, 23.15)	20.42 (18.15, 23.09)	0.610
SpO^2 mean	Median (IQR)	95.84 (94.08, 97.46)	96.31 (94.64, 98.00)	0.006

Glucose mean (mg/dL)	Median (IQR)	139.44 (114.50, 181.00)	130.50 (109.00, 160.00)	0.013
**Laboratory Results**				
PH min	Median (IQR)	7.30 (7.22, 7.36)	7.28 (7.22, 7.35)	0.234
PH max	Median (IQR)	7.39 (7.35, 7.43)	7.39 (7.35, 7.43)	0.899
PO^2 min	Median (IQR)	42.00 (32.00, 59.00)	38.50 (31.00, 66.00)	0.700
PO^2 max	Median (IQR)	91.00 (56.00, 160.00)	110.00(66.00, 208.00)	0.016
PCO^2 min	Median (IQR)	42.00 (35.00, 51.00)	43.50 (36.00, 49.00)	0.984
PCO^2 max	Median (IQR)	55.00 (45.00, 69.00)	55.00 (48.00, 66.00)	0.428
BE min	Median (IQR)	0.00 (-5.00, 2.00)	-1.00 (-5.00, 1.00)	0.279
BE max	Median (IQR)	1.00 (-1.00, 5.00)	1.00 (-1.00, 4.00)	0.732
Total CO^2 min	Median (IQR)	26.00 (22.00, 31.00)	26.00 (21.00, 30.00)	0.712
Total CO^2 max	Median (IQR)	29.00 (25.00, 34.00)	30.00 (26.00, 34.00)	0.510
Scr min	Median (IQR)	0.80 (0.60, 1.30)	0.80 (0.60, 1.40)	0.578
Scr max	Median (IQR)	148.00 (145.00, 150.00)	150.00 (147.3, 150.00)	<0.001
GFR	Median (IQR)	0.81 (0.79, 1.03)	1.00 (0.79, 1.03)	0.470
Platelets min	Median (IQR)	178.00 (130.00, 241.00)	178.50(137.00, 227.00)	0.777
Platelets max	Median (IQR)	209.00 (155.00, 281.00)	217.00 (160.00, 271.00)	0.859
Wbc min	Median (IQR)	10.20 (7.50, 14.00)	10.30 (7.30, 12.70)	0.238
Wbc max	Median (IQR)	13.40 (9.50, 18.10)	13.30 (9.70, 17.00)	0.541
AG min	Median (IQR)	13.00 (11.00, 16.00)	13.00 (11.00, 15.00)	0.631
AG max	Median (IQR)	17.00 (14.00, 20.00)	16.50 (14.00, 19.00)	0.994
HCO^3 min	Median (IQR)	23.00 (19.00, 26.00)	22.00 (19.00, 26.00)	0.569
HCO^3 max	Median (IQR)	25.00 (22.00, 29.00)	26.00 (23.00, 29.00)	0.858
Bun min	Median (IQR)	24.00 (17.00, 40.00)	28.00 (18.00, 47.00)	0.064
Bun max	Median (IQR)	29.00 (20.00, 46.00)	35.50 (22.00, 54.00)	0.021
Calcium min	Median (IQR)	8.30 (7.80, 8.80)	8.30 (7.70, 8.70)	0.632
Calcium max	Median (IQR)	8.70 (8.30, 9.20)	8.75 (8.20, 9.20)	0.954
INR min	Median (IQR)	1.20 (1.10, 1.40)	1.20 (1.10, 1.40)	0.654
INR max	Median (IQR)	1.30 (1.10, 1.60)	1.30 (1.10, 1.60)	0.746

Pt min	Median (IQR)	13.20 (11.90, 15.30)	13.25 (11.80, 15.60)	0.625
Pt max	Median (IQR)	13.90 (12.40, 17.20)	14.00 (12.50, 17.30)	0.714
Ptt min	Median (IQR)	29.20 (26.20, 33.50)	28.65 (26.10, 32.80)	0.420
Ptt max	Median (IQR)	32.60 (28.70, 45.80)	32.45 (28.10, 42.20)	0.545
Hematocrit min	Median (IQR)	31.00 (25.60, 35.80)	30.60 (26.70, 35.50)	0.880
Hematocrit max	Median (IQR)	34.60 (29.30, 39.20)	34.45 (29.60, 39.50)	0.760
Hemoglobin min	Median (IQR)	9.70 (8.10, 11.30)	9.45 (8.20, 11.30)	0.919
Hemoglobin max	Median (IQR)	10.80 (9.00, 12.30)	10.80 (9.30, 12.70)	0.573
Urine Output	Median (IQR)	1276.00 (740.00, 2140.00)	1227.50 (765.00, 1850.00)	0.226

GCS: Glasgow Coma Scale; CKD: Chronic Kidney Disease; HFNC: High-flow Nasal Cannula
