# Supplementary material for: Enhanced machine learning predictive modeling for delirium in elderly ICU patients with COPD and respiratory failure: A retrospective study based on MIMIC-IV
Source: PLoS One. 2025 Mar 20;20(3):e0319297. doi: 10.1371/journal.pone.0319297 (PMC11925466; doi:10.1371/journal.pone.0319297)
Supplement: S1 File — (PDF) [file pone.0319297.s002.pdf]

Variable screening process (Lasso with optimal subset regression)

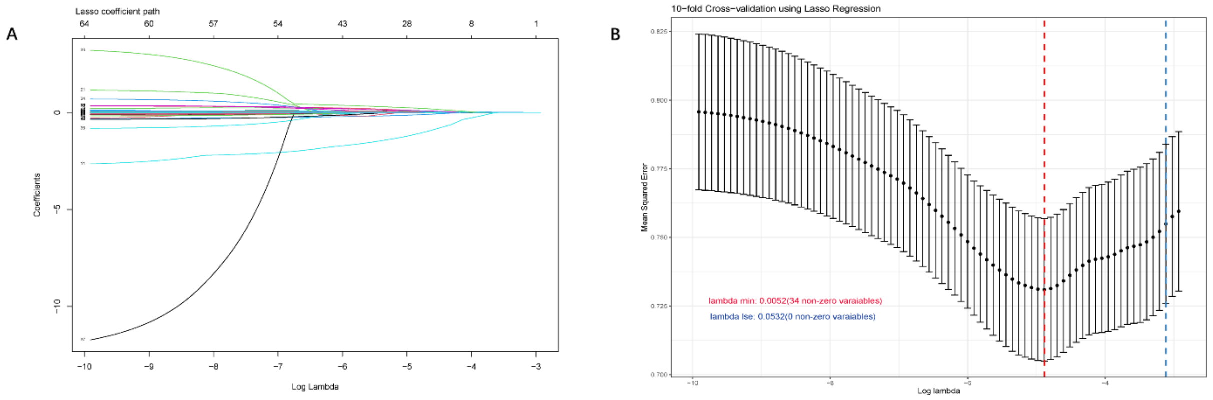

Fig1 Lasso regression (A) Lasso regression path diagram, which shows how the model coefficients change under different levels of penalties. (B) Lasso cross-validation plots, the α value corresponding to the highest cross-validation score or the smallest mean square error is chosen as the optimal parameter.

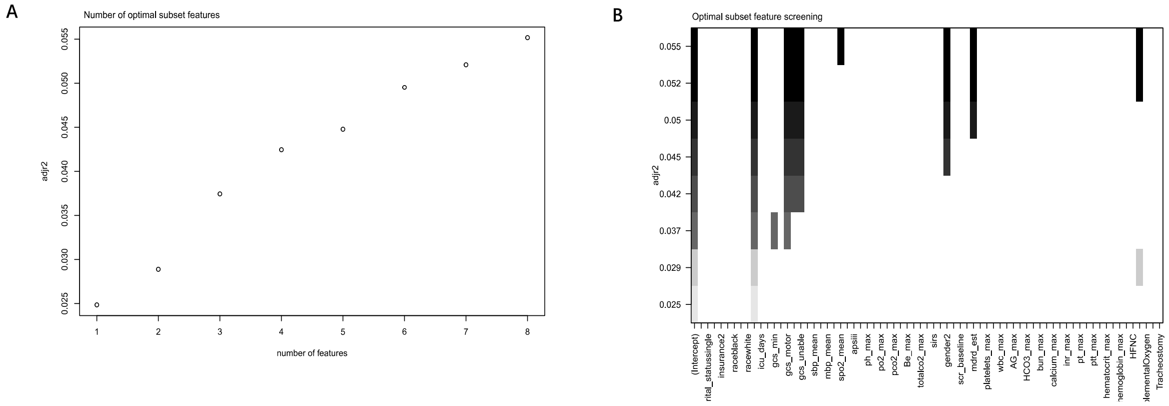

Fig2 Best subset regression. (A)Number of optimal subset features point plot based on Adjust R^2. (B) Comparison chart based on Adjust R^2 optimal subset models.
